# Long-term results of glaucoma drainage device surgery

**DOI:** 10.1186/s12886-019-1027-z

**Published:** 2019-01-10

**Authors:** Konstantine Purtskhvanidze, Mark Saeger, Felix Treumer, Johann Roider, Bernhard Nölle

**Affiliations:** Department of Ophthalmology, University Medical Center Schleswig-Holstein, Arnold-Heller Strasse 3, Haus 25, D-24105 Kiel, Germany

**Keywords:** Ahmed valve, Baerveldt implant, Glaucoma surgery, Glaucoma drainage device, IOP control

## Abstract

**Background:**

To evaluate long-term results of eyes with glaucoma drainage device (GDD).

**Methods:**

We retrospectively reviewed medical records of all patients who underwent GDD placement at our institution between 2001 and 2014. A total of 110 eyes of 90 patients were studied. Glaucoma outcome was assessed by postoperative intraocular pressure (IOP), number of medications, and need for further glaucoma surgery. Surgical procedures before and during the study period, and their complications were evaluated.

**Results:**

The mean follow-up was 78.3 ± 44.0 months. The mean preoperative intraocular pressure was 30.8 ± 6.9 mmHg with 3.5 ± 1.1 glaucoma medications. At last postoperative follow-up, the mean IOP decreased to 14.3 ± 5.4 mmHg with 1.6 ± 1.5 glaucoma medications. GDD implantation successfully controlled glaucoma in 86, 85, 81, 78, 79, 76 and 73% of eyes at 1, 2, 3, 4, 5, 7 and 10 years, respectively. At last follow-up IOP was successfully controlled in 67% of eyes. Clinical complications occurred in 56.4% of eyes during the follow-up period.

**Conclusions:**

A glaucoma drainage device can successfully control intractable glaucoma even after a very long period of time.

## Background

Glaucoma is a leading cause of blindness worldwide [1]. Intraocular pressure (IOP) lowering is the only proven method to prevent the development [2] and/or slow the progression of glaucomatous optic neuropathy [3]. The conventional approach is to attempt medical therapy or laser trabeculoplasty prior to surgery.

Recently, the use of glaucoma drainage devices (GDDs) has become a widely used therapy option for the management of eyes with complicated glaucoma refractory to standard trabeculectomy with adjunctive antifibrotics. This trend is following the results of the Tube Versus Trabeculectomy study, which reported a better success rate at 5 years with Baerveldt implantation than with trabeculectomy with mitomycin-C in patients who had undergone previous surgery [4].

Since Molteno’s introduction in 1969 different modified types of drainage devices, which differ in surface area, shape, composition, and presence or absence of a flow-restricting valve are currently used [5]. Two of the most common devices are the valved Ahmed Glaucoma Valve (AGV; New World Medical, Rancho Cucamonga, CA, USA) and the non-valved Baerveldt Glaucoma Implant (BGI; Abbott Medical Optics, Abbott Park, IL, USA). These devices have been shown to be effective in lowering IOP for the treatment of glaucoma in patients with a variety of glaucomas in whom medical therapy or multiple trabeculectomies have failed or are expected to have a very low chance of surgical success [6–9]. The surgical success rates are dependent on the length of follow-up, success criteria, and different types of glaucoma. They vary from 68 to 100% for the Ahmed Glaucoma Valve and from 43 to 100% for the Baerveldt Glaucoma Implant [5].

In this study, we present long-term follow-up results of eyes with glaucoma drainage device. In addition, all relevant clinical factors were evaluated for their association with glaucoma outcome.

## Methods

The medical records of all patients who underwent GDD placement (Baerveldt or Ahmed) at the Department of Ophthalmology of the University Medical Center Schleswig-Holstein Campus Kiel between 2001 and 2014 were retrospectively reviewed. Patients who underwent a penetrating keratoplasty prior to GDD implantation were excluded.

One hundred ten eyes of 90 patients were included in this study. Study criteria required a minimum of 2-year follow-up. Collected data included demographic information, type of glaucoma, type of corneal pathology, number and type of previous ophthalmic surgeries, complications, preoperative and postoperative best-corrected visual acuity, preoperative and postoperative IOP, number and type of glaucoma medications. Surgical outcome was assessed in terms of adequacy of IOP control. Success for glaucoma control was defined as a postoperative IOP ≥ 5 mmHg and ≤ 21 mmHg with or without application of antiglaucoma medications, with no need for further glaucoma surgery and without loss of light perception at last follow-up. Combination medication eye drops were counted as 2 medications. An oral carbonic anhydrase inhibitor was counted as 1 additional medication. Visual acuity was measured with the Snellen vision chart. For numerical analysis, Snellen visual acuity was converted to logarithm of the minimum angle of resolution (logMAR) values using the equitation: logMAR = ─log (visual fraction).

### Surgical technique

All glaucoma surgical procedures were carried out by the same experienced surgeon (B. N.) of the Department of Ophthalmology. GDDs used in this study were Ahmed™ Glaucoma Valve (FP7 AGV; New World Medical, Rancho Cucamonga, CA, USA) and Baerveldt (250 mm^2^ BGI; Abbott Medical Optics, Abbott Park, IL, USA). The surgical techniques of GDD implantation have been decribed in detail by others before [10, 11]. Briefly, a limbus based conjunctival peritomy was done with a blunt dissection down to bare sclera in 2 to 3 quadrants. Implants were placed between the lateral rectus and the superior rectus if possible. For Baerveldt implants a 7–0 vicryl ligature suture was placed to form a watertight seal around the tube. GDD plates were secured to sclera with 7–0 prolene sutures. A limbal tract was created with a 23-gauge needle. Then a prompt insertion of the tube through insertion forceps was performed. Tubes were inserted into the anterior chamber just overlying the iris without touching the cornea. In eyes where the tube was positioned in the pars plana, a pars plana vitrectomy (PPV) was performed. A scleral fistula was created 3.0 to 3.5 mm posterior to the corneoscleral limbus using a 22- or 23-gauge needle directed parallel to the iris plane. In cases of anterior chamber tube positioning the tube was placed within the sclera, for about one third of its length. Therefore no xenogeneic or other tissue was used for coverage. Tubes were secured to the sclera with 1–3 interrupted 10–0 nylon sutures. The conjunctiva and Tenon’s tissue were sutured in single layer using 8–0 vicryl sutures. At the end of GDD surgery no leakage was observed in any of the eyes. A subconjunctival injection of dexagentamicin was given at the end of surgery. Postoperative topical medications included antibiotics and corticosteroids depending on the degree of intraocular inflammation. The medication was tapered to a low dose over several months. If necessary, glaucoma medications were added to control IOP.

### Statistical analysis

The statistical analyses were performed using the SPSS software (IBM SPSS Statistics, Version 24). Univariate analysis was performed using the log-rank test for comparing Kaplan-Meier survival curves. For preoperative and postoperative analyses, the Wilcoxon signed-rank test, and the Wilcoxon rank sum test (U-test) were used for measured data. The Fisher exact test was used for nominal scaled data. A *p*-value less than 0.05 was considered statistically significant.

## Results

A total of 110 eyes of 90 patients were included. The demographic data and preoperative clinical characteristics of the study patients are presented in Table 1. The mean patient age was 47.9 ± 24.4 years (range, 2–87). The mean follow-up was 78.3 ± 44.0 months (range, 24–193). Glaucoma diagnoses were divided into different types.

**Table 1:** Patient Demographic Data

Number of Patients	90
Number of Eyes	110
Age range (mean ± SD), y	2–87 (47.9 ± 24.4)
Sex	
Male	35/90 (38.9)
Female	55/90 (61.1)
Glaucoma type	
Primary open angle	50/110 (45.5)
Chronic angle closure	1/110 (0.9)
Congenital	18/110 (16.4)
Uveitic	27/110 (24.6)
Pseudoexfoliative	5/110 (4.5)
Traumatic	2/110 (1.8)
Iridocorneal endothelial Syndrom	2/110 (1.8)
Aphakia	4/110 (3.6)
Pigmentary	1/110 (0.9)
Pre-GDD lens status	
Aphakic	17/110 (15.5)
Phakic	35/110 (31.8)
PCIOL	58/110 (52.7)
Ocular surgeries pre-GDD	
Range (mean ± SD)	1–8 (2.7 ± 1.4)
Trabeculectomy	92/110 (83.6)
Phaco/PCIOL	57/110 (51.8)
Pas plana vitrectomy	12/110 (10.9)
Trabeculectomy revision	14/110 (12.7)
Anterior vitrectomy, lensectomy	13/110 (11.8)
Diode cyclophotocoagulation	31/110 (28.2)
Cyclocryo	7/110 (6.4)
Scleral buckle procedure	1/110 (0.9)

*GDD* glaucoma drainage device, *PCIOL* posterior chamber intraocular lens

One hundred one (91.8%) Ahmed implants and 9 (8.2%) Baerveldt implants were used. Eighty-three (75.5%) implants were placed in the anterior chamber, and 27 (24.5%) implants were positioned in the pars plana. The number of performed surgical procedures before the study period was 2.7 ± 1.4 (Table 1). During the study period the number of additionaly performed surgical procedures was 1.5 ± 1.4 (Table 3).

### Glaucoma outcome

**Fig. 1:**
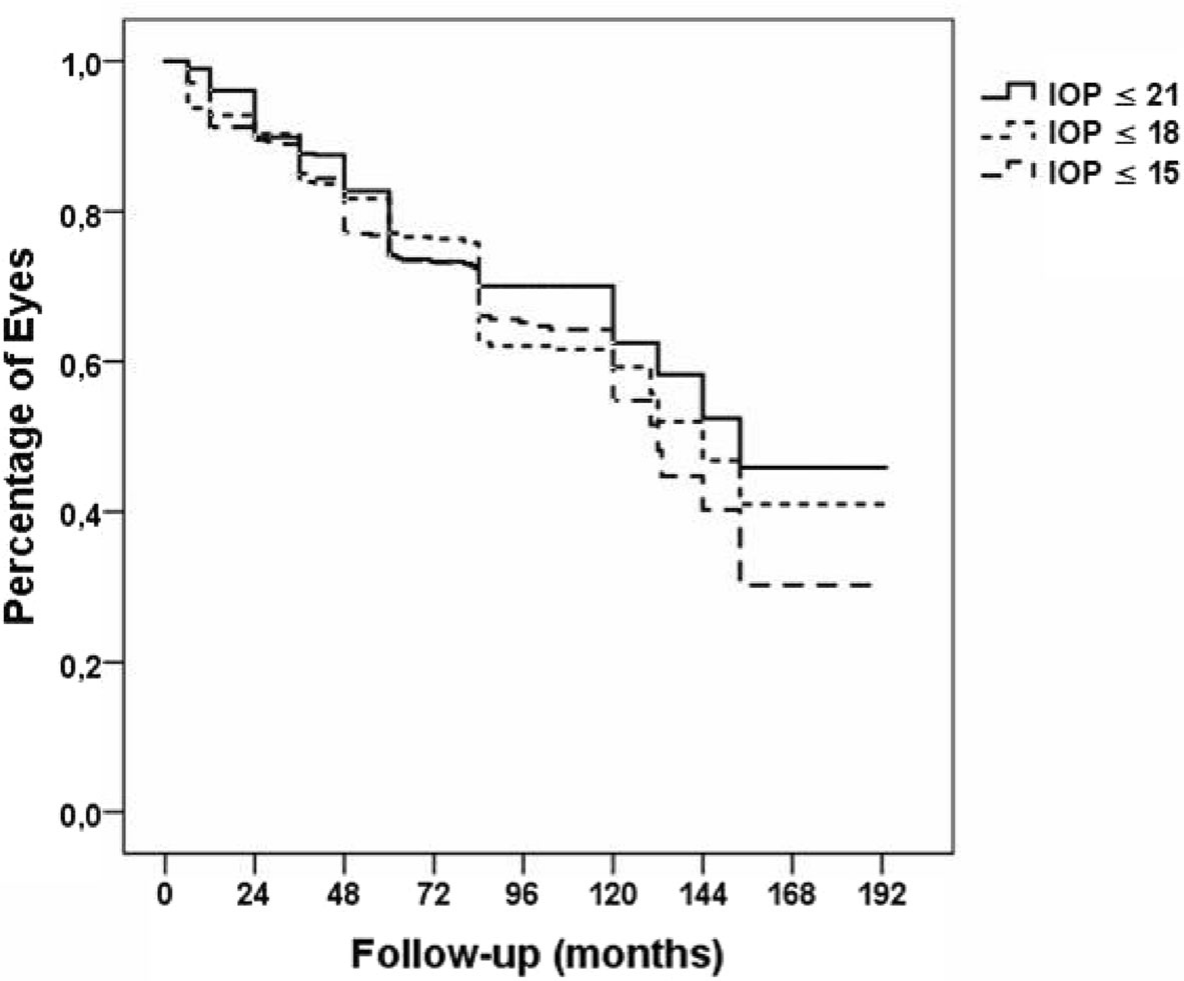
Kaplan-Meier survival analysis of IOP (intraocular pressure)

The mean preoperative intraocular pressure was 30.8 ± 6.9 mmHg. At last postoperative follow-up, the mean IOP had decreased to 14.3 ± 5.4 mmHg (*p* = 0.001) (Fig. 1). The mean preoperative glaucoma medications were 3.5 ± 1.1. The mean glaucoma medications were 1.1 ± 1.2, 1.1 ± 1.2, 1.5 ± 1.3, 1.6 ± 1.4, 1.9 ± 1.6, 1.8 ± 1.7, 1.8 ± 1.6 at 1, 2, 3, 4, 5, 7 and 10 years, respectively. At last postoperative follow-up, the mean glaucoma medications were 1.6 ± 1.5 (*p* = 0.001) (Table 2). Overall, GDD implantation successfully controlled glaucoma in 86, 85, 81, 78, 79, 76 and 73% of eyes at 1, 2, 3, 4, 5, 7 and 10 years, respectively. At last follow-up IOP was successfully controlled in 67% of eyes. The success rate of the Ahmed implant was 68%, and of the Baerveldt implant 56% at last follow-up. However, this difference was not statistically significant (*p* = 0.47).

**Table 2:** Postoperative Data

Follow-up (mo)	
Mean ± SD (range)	78.3 ± 44.0 (24–193)
Total Number of eyes at each follow-up	
2 yrs	110
3 yrs	92
4 yrs	80
5 yrs	68
7 yrs	52
10 yrs	23
Pre-GDD IOP (mmHg)	
Mean ± SD (range)	30.8 ± 6.9 (20–52)
IOP at last follow-up visit (mmHg)	
Mean ± SD (range)	14.3 ± 5.4 (2–34)
Pre-GDD glaucoma medications (number)	
Mean ± SD (range)	3.5 ± 1.1 (1–5)
Medications at last follow-up (number)	
Mean ± SD (range)	1.6 ± 1.5 (0–5)
Pre-GDD VA	
Mean ± SD	0.7 ± 0.6
VA at last follow-up visit	
Mean ± SD	0.9 ± 0.7

*GDD* glaucoma drainage device, *IOP* intraocular pressure, *VA* visual acuity

**Fig. 2:**
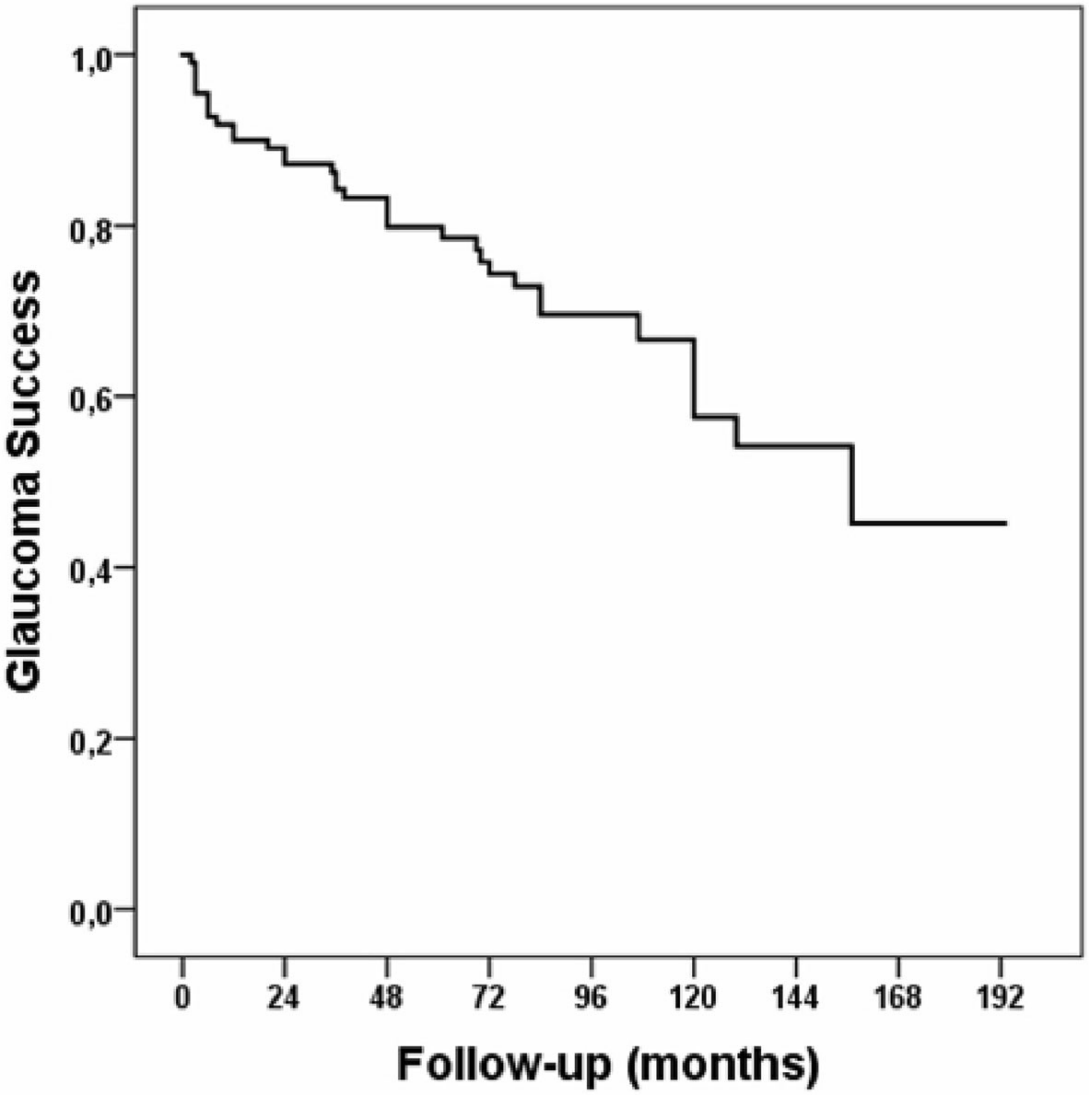
Overall Kaplan-Meier survival analysis of glaucoma success throughout follow-up

In terms of tube placement, the glaucoma success rate of the anterior chamber tube placement was 64%, and of the pars plana tube placement 78% at last follow-up. No statistically significant difference in success rates was observed (*p* = 0.56). The overall survival of glaucoma outcome was analyzed with a Kaplan-Meier curve (Fig. 2).

### Visual acuities

Each patient’s preoperative visual acuity was compared with the visual acuity at the last follow-up. The mean pre-GDD visual acuity (in logMAR) was 0.7 ± 0.6. At last postoperative follow-up, the mean visual acuity (logMAR) decreased to 0.9 ± 0.7 (*p* = 0.002) (Table 2). In a comparision of visual acuitiy between first and last visits, 33 (30%) eyes showed improvement, 20 (18%) eyes had no change, and 57 (52%) worsened. Of these 57 eyes where the visual acuity decreased, 20 (35%) eyes had a corneal decompensation.

### Complications

Clinical complications occurred in 62 (56.4%) eyes during the follow-up period. Postoperative complications included corneal decompensation (*n* = 20, 19%), encapsulated bleb (*n* = 27, 24.5%), retinal detachment (*n* = 7, 6.4%), GDD or tube dislocation (*n* = 7, 6.4%), tube erosion (*n* = 7, 6.4%), tube-endothelial touch or blockage (*n* = 5, 4.5%), chronic hypotony (*n* = 5, 4.5%), and phthisis bulbi (*n* = 4, 3.6%). Table 3 shows complications during follow-up. Overall, the rate of complications was not dependent on the tube placement (*p* = 0.5).

**Table 3:** Additional Procedures and Complications

GDD type	N (%)
Ahmed FP7	101/110 (91.8)
Baerveldt 250 mm^2^	9/110 (8.2)
Location for tube placement	
Anterior chamber	83/110 (75.5)
Pars plana	27/110 (24.5)
Operative and post-GDD complications	
Corneal decompensation	20/110 (19.0)
Retinal detachment	7/110 (6.4)
Encapsulated bleb	27/110 (24.5)
GDD dislocation	5/110 (4.5)
Tube erosion	7/110 (6.4)
Tube dislocation	2/110 (1.8)
Tube-endothelial touch	3/110 (2.7)
Tube blockage	2/110 (1.8)
Chronic hypotony^a^	5/110 (4.5)
Phthisis bulbi	4/110 (3.6)
No light perception	4/110 (3.6)
Patients with complications	62/110 (56.4)
Additional post-GDD procedures	
Mean ± SD (range)	1.5 ± 1.4 (0–6)
Revision of existing GDD	2/110 (1.8)
Tube shortening/revision	16/110 (14.5)
Tube coverage	5/110 (4.5)
Drainage of choroidal effusion	5/110 (4.5)
Implantation of additional GDD	12/110 (10.9)
GDD explantation	3/110 (2.7)
Diode cyclophotocoagulation	17/110 (15.4)
Phaco/PCIOL	9/110 (8.2)
Pars plana vitrectomy	10/110 (9.1)
Scleral buckle procedure	1/110 (0.9)
Needling of filtering bleb/5*-*FU injections	27/110 (24.5)
Healon®-injection (anterior chamber)	26/110 (23.6)
DSAEK/PKP	20/110 (19.0)
Enucleation	1/110 (0.9)

^a^IOP < 5 mmHg for ≥3 months

*GDD* glaucoma drainage device, *PCIOL* posterior chamber intraocular lens, *DSAEK* descemet’s stripping automated endothelial keratoplasty, *5-FU* 5*-*fluorouracil, *PKP* penetrating keratoplasty

**Fig. 3:**
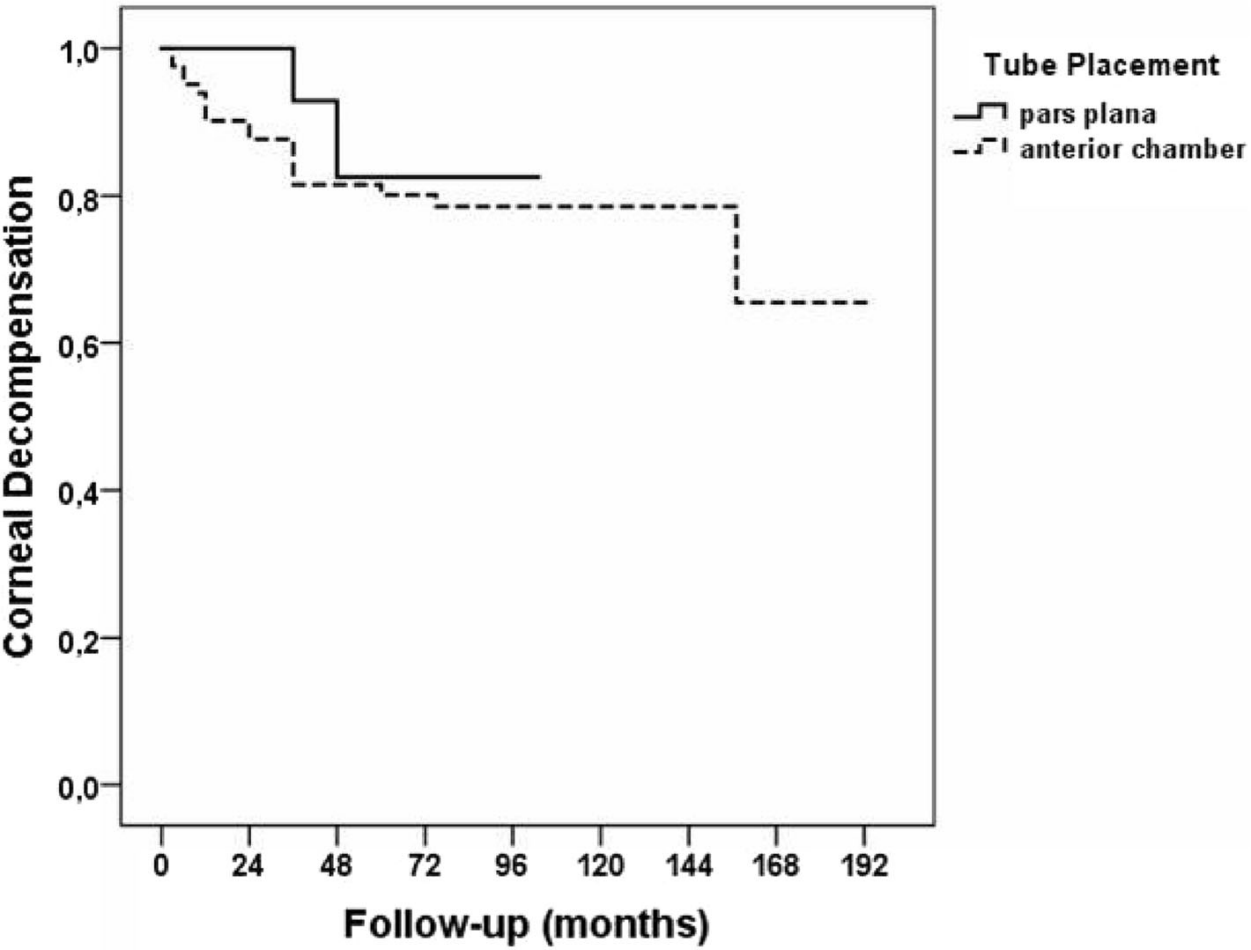
Kaplan-Meier survival analysis of corneal clarity throughout follow-up

Five eyes had a corneal decompensation prior to GDD implantation. That is why these eyes were excluded from the statistical analysis of postsurgical corneal decompensation (2 eyes with anterior chamber tube placement, and 3 eyes with pars plana tube placement). During follow-up period corneal decompensation ocurred in 18 of 81 eyes (22%) with anterior chamber tube placement, and only in 2 of 24 eyes (8%) with pars plana tube placement (*p* = 0.15). At 2-year follow-up, there was a trend towards increased corneal decompensation in eyes with anterior chamber tube placement compared to pars plana tube placement (*p* = 0.076). From the third year of the study, no difference was observed between both groups (*p* = 0.14) (Fig. 3). Due to the fact that eyes with pars plana tube placement had a shorter follow-up (up to 108 months), we only compared the corneal decompensation associated with tube placement over a shorter period.

### Surgical procedures during study period

During the study period the number of additional surgical procedures was 1.5 ± 1.4 (Table 3). The most commonly performed additional glaucoma procedures during the follow up period were needling of filtering bleb/5*-*FU injections (*n* = 27), keratoplasty (*n* = 20), tube shortening/revision (*n* = 16), and implantation of additional GDD (*n* = 12). Overall, reoperations for complications during the follow-up were not dependent on the tube placement (*p* = 0.16). Surgical complications an additional glaucoma procedures are shown in Table 3.

## Discussion

Glaucoma drainage devices are commonly used to lower IOP in patients with difficult glaucomas either as a primary surgical option or after failure of conventional filtration procedures [12, 13]. A growing experience with these devices, improvements in the material design and surgical techniques for performing GDD implantation have also led to increased utilization in recent years [14]. Data of recently published studies, which observed the efficacy of GDDs contribute to a paradigm shift in glaucoma treatment. The TVT study shows a persistent treatment benefit of tube shunt surgery over trabeculectomy through 5 years of follow-up. At 5 years, the cumulative probability of failure was 29.8% in the tube group and 46.9% in the trabeculectomy group [4]. The results from our study demonstrated overall a cumulative probability of glaucoma failure of 21% through 5 years of follow-up. In contrast to the TVT study, which used Baerveldt implants, in our series only 9 patient received a Baerveldt implant. All other patients had an Ahmed implantation. Due to the fact that our study did not include enough patients with Baerveldt implants, it was difficult to compare the efficacy of both implants.

Three year results of the ABC study [6] demonstrated a cumulative probability of failure of 31.3% in the AGV group, and of 32.1% in the BGI group. At 3 years, the AVB study [9] reported a failure rate of 51% in the AGV group and of 34% in the BGI group. In our study, patients who received the Ahmed implant showed a failure rate of 20%, patients who received the Baerveldt implant showed a failure rate of 11% after a 3 year follow-up. The cumulative probabilty of failure for both implants was 19% at 3 year follow-up.

At 5 years of follow-up, the ABC study observed a cumulative probability of failure of 44.7% in the AGV group, and of 39.4% in the BGI group [6]. In our study, patients who received the Ahmed implant showed a failure rate of 22%, patients who received the Baerveldt implant showed a failure rate of 11% after a 5 year follow-up. The cumulative probabilty of failure for both implants was 21% at 5 year follow-up. At last follow-up of 78.3 ± 44.0 months, a failure of glaucoma outcome occurred in 32% of eyes.

Rososinski et al. evaluated outcomes of pars plana versus anterior chamber placement of Baerveldt implants (anterior chamber tube placement in 34 eyes, pars plana tube placement in 29 eyes) [15]. They reported a qualified success rate at 2 years of 94% for the pars plana group and of 91% for the anterior chamber group [15]. In our study (anterior chamber tube placement in 83 eyes, pars plana tube placement in 27 eyes), the success rate at 2 years was 78% for the pars plana group, and 88% for the anterior chamber group (*p* = 0.22).

Guzman et al. examined the outcomes of glaucoma drainage devices (33 eyes, Molteno and Baerveldt tubes) inserted into the pars plana. They described a qualified success rate of 42.4% at 30 months [16]. In our study (27 eyes with pars plana tube placement), the success rate at 3 years was 78%. At last follow-up of 78.3 ± 44.0 months, our results of eyes with tubes placed in the pars plana showed IOP control in 78%, and in eyes with anterior chamber tube placement in 64%, respectively (*p* = 0.56).

Christiakis et al. reported a postoperative complication rate of 52% at 3 year follow-up [9]. At last follow-up, we found a postoperative complication rate of 56%. Rososinski et al. described no case of corneal decompensation or corneal graft failure in eyes with pars plana Baerveldt tube implantation during their 2 year follow-up period [15]. The results from our study demonstrated a corneal decompensation in 1 of 27 eyes (4%) with pars plana Baerveldt or Ahmed tube implantation. Souza et al. experienced the complication of failed penetrating corneal keratoplasty during the 5 year follow-up period in 5% (4 eyes) after anterior chamber Ahmed implantation [17]. In our study, at 5 years follow-up corneal decompensation occured in 19%. Overall in our study, no difference was observed between eyes with anterior chamber tube placement compared to pars plana tube placement. Eyes with pars plana tube placement had a shorter follow-up. Therefore, we could only compare the tube placement over a shorter period.

There are some limitations to our study. The major limitation is its retrospective design. Further limitations are the inclusion of patients with various glaucoma diagnoses and variable severity of disease, variety of used GDDs, and different locations for the tube placement.

However, it is important to note that the sample size of this study is comparatively large. Furthermore, all surgical procedures were performed by the same surgeon. This study has a remarkably long follow*-*up period*,* which allows an assessment of long-term outcome.

## Conclusions

Our study provides long-term follow-up outcomes of patients with glaucoma drainage devices. Overall, our data show that the glaucoma success with the tube implant was high. A glaucoma drainage device placement can provide effective glaucoma control even after a very long period of time.

## Abbreviations

5-FU: 5*-*fluorouracil
ABC study: Ahmed Baerveldt Comparison Study Group
AGV: Ahmed glaucoma valve
AVB study: Ahmed versus Baerveldt study
BGI: Baerveldt glaucoma implant
DSAEK: Descemet’s stripping automated endothelial keratoplasty
GDD: Glaucoma drainage device
IOP: Intraocular pressure
logMAR: Logarithm of the minimum angle of resolution
mmHg: Millimetre (s) of mercury
PCIOL: Posterior chamber intraocular lens
PKP: Penetrating keratoplasty
ppV: Pars plana vitrectomy
TVT study: Tube Versus Trabeculectomy study
VA: Visual acuity
